# A meta‐analysis of the hamstring tendon strands reconstruction in ACL: Functional outcomes based on strands number

**DOI:** 10.1002/jeo2.70508

**Published:** 2025-11-03

**Authors:** Juan M. Fernández‐Domínguez, José L. Martín‐Alguacil, Marta Esteban‐Blanco, Manuel Vides‐Fernández, Joan Carles Monllau

**Affiliations:** ^1^ Clinica Martin Gomez, Vithas Traumatology Service Granada Spain; ^2^ Institute of Health Sciences Studies Foundation of Castilla y León Health Training and Research Traumatology Service León Spain; ^3^ Hospiten Estepona Traumatology Service Málaga Spain; ^4^ Hospital parc del Mar Traumatology Service Barcelona Spain

**Keywords:** anterior cruciate ligament, hamstring, outcomes, reconstruction, strand

## Abstract

**Purpose:**

Comparing the effectiveness and safety of four‐, five‐, six‐ and eight‐strand hamstring tendon graft topologies for anterior cruciate ligament (ACL) restoration was the aim of this meta‐analysis. In order to provide evidence‐based recommendations for graft selection, the study sought to assess clinical and functional results, including graft diameter, postoperative stability, graft failure rates, and patient‐reported functional outcomes.

**Methods:**

This systematic review used the PICOS methodology and adhered to PRISMA standards. Randomized controlled trials and cohort studies contrasting four‐strand hamstring grafts with alternative designs for ACL restoration were among the studies that qualified. The databases PubMed, EMBASE, SCOPUS and Cochrane were searched extensively for relevant material. Independent data extraction was done, and disagreements were settled by consensus. Graft diameter, patient‐reported scores (Lysholm, IKDC), stability (as determined by KT‐1000/2000 arthrometers), and graft failure rates were among the results. Heterogeneity‐level‐based fixed‐ and random‐effects models were used in the statistical study.

**Results:**

Eight studies with 1161 participants were analyzed. Multi‐strand grafts (five‐ and six‐strands) demonstrated increased graft diameters and superior stability compared to four‐strand grafts. The four‐strand configuration had significantly lower Lysholm and Tegner scores and smaller graft diameters. However, no significant differences in IKDC scores or revision rates were found between four‐ and five‐strand groups. Clinical tests (Lachman, pivot‐shift) showed no significant differences across graft types.

**Conclusion:**

Multi‐strand hamstring grafts (≥five strands) offer advantages, such as larger graft diameters and improved stability, over four‐strand grafts. However, these benefits did not consistently translate into superior patient‐reported outcomes or lower revision rates. The success of ACL reconstruction is multifactorial, involving graft selection, surgical techniques, and individual patient factors. Further studies are necessary to optimize graft configuration and long‐term outcomes.

**Level of Evidence:**

Level III.

**Abbreviations**
ACL: anterior cruciate ligament
CI: confidence interval
GRADE: Grading of Recommendations Assessment, Development and Evaluation
IKDC: International Knee Documentation Committee
KT: knee test
MCS: mental component summary
MD: mean difference
MINORS: methodological index for non‐randomized studies
OR: odds ratio
PCS: physical component summary
PICOS: population intervention comparison outcomes and study design
PRISMA: preferred reporting items for systematic reviews and meta‐analyses
PROMS: patient‐reported outcome measures
SF‐36: Short form 36
SMD: standardized mean difference

## INTRODUCTION

Anterior cruciate ligament (ACL) injuries are among the most common sports‐related knee injuries and often require surgical reconstruction to restore joint stability and function. Because of their good long‐term results, low donor site morbidity, and advantageous characteristics, hamstring tendon autografts are a popular choice for ACL restoration. However, despite their widespread use, there remains significant variability in surgical technique, particularly regarding the number of strands used to construct the graft. The most widely used designs are four‐strand (quadruple) grafts, although as surgeons work to maximize graft diameter and mechanical strength, there is growing interest in five‐, six‐ and eight‐strand constructs [14].

Graft diameter has been identified as a critical factor influencing the risk of graft failure, particularly in young and highly active patients. Previous studies have demonstrated that grafts measuring less than 8 mm in diameter are associated with significantly higher rates of rerupture. Some surgeons have responded to this by using multi‐strand designs, arguing that more strands could produce thicker grafts with higher load‐bearing capability. In fact, compared to conventional four‐strand structures, five and six strang‐satrand grafts have demonstrated to be able to attend (obteber) a higher ultimate failure stresses. These theoretical advantages, however, have not consistently translated into superior clinical outcomes [11].

Although multi‐strand grafts may demonstrate increased tensile strength in vitro, the impact of these configurations on postoperative stability, graft survival, and patient‐reported functional outcomes remains uncertain. Furthermore, surgical time, technical requirements and the viability of graft preparation must all be taken into consideration when making the clinical choice to enhance graft complexity [2]. The existing literature includes heterogeneous studies with variable designs, populations, follow‐up durations, and outcome measures, resulting in inconclusive and often conflicting findings.

Given this lack of consensus, there is a clear need to systematically evaluate the current evidence comparing different hamstring tendon graft configurations. To guide surgeons choose the best and most suitable graft build, especially in high‐demand patients where long‐term graft integrity is critical, it is imperative to comprehend if the inclusion of additional strands gives significant therapeutic benefits.

Moreover, the purpose of this systematic review and meta‐analysis is to assess the safety and efficacy of different hamstring tendon graft configurations—specifically four‐, five‐, six‐ and eight‐strand constructs—in ACL reconstruction. To ascertain whether adding more strands yields better results, we looked at important outcomes such as graft diameter, objective postoperative knee stability, graft failure rates and patient‐reported functional outcomes.

The hypothesis of this study is that the use of four‐strand grafts in ACL reconstruction is associated with smaller graft diameters and inferior patient‐reported functional outcomes compared to grafts composed of a higher number of strands, without a corresponding increase in objective knee laxity or graft failure rates. By clarifying the clinical relevance of different graft configurations, this study aims to inform surgical decision‐making and contribute to the optimization of ACL reconstruction techniques.

## MATERIALS AND METHODS

### Eligibility criteria

This systematic review and meta‐analysis followed PRISMA guidelines to ensure transparency and reproducibility [13].

The PICOS (Population, Intervention, Comparison, Outcomes, Study design) framework were used to define the eligibility criteria for this systematic review and meta‐analysis. The population (P) included patients who underwent primary ACL reconstruction surgery. The intervention group (I) consisted of patients who underwent reconstruction using a four‐strand hamstring tendon graft. The comparison group (C) comprised of patients treated with different strands for ACL reconstruction. The primary outcomes of interest (O) were the efficacy and safety of each strand type. Only comparative study designs (S) were considered eligible for inclusion, specifically randomized controlled trials, prospective cohort studies, and retrospective cohort studies, in order to minimize selection and information biases.

Studies were excluded from the meta‐analysis if they met one or more of the prespecified criteria to ensure that only the most robust comparative evidence was included. Duplicates were excluded to avoid overrepresentation and redundancy of the data from the same study population. Noncomparative studies such as case reports, case series and letters to the editor were excluded because they lacked a control group for uncontrolled treatment comparisons. Trial protocols were excluded as they did not report the implemented study results. Studies with incomplete or noncomparable outcome data across the treatment groups were excluded to facilitate pooling and meaningful statistical comparisons in the meta‐analysis.

### Inclusion criteria

Comparative studies evaluating primary ACL reconstruction using hamstring tendon grafts were included, comparing four‐strand configurations with grafts using a higher number of strands (five, six or eight). Eligible studies included randomized clinical trials, as well as prospective and retrospective cohort studies, that reported at least one of the following outcomes: graft diameter, objective knee laxity (measured with KT‐1000/2000), patient‐reported outcome measures (PROMs), graft failure rates, or clinical tests such as the Lachman, pivot‐shift or hoop test. A minimum follow‐up of 6 months and the availability of comparable data between groups were required. Excluded were duplicate studies, noncomparative studies, protocols without results, reports involving patient groups with nonequivalent characteristics or lacking useful clinical data, and studies with incomplete or inaccessible information.

### Information sources

A comprehensive literature search was conducted in PubMed, EMBASE, SCOPUS and the Cochrane Library with no restrictions on language or date of publication. In addition, reference lists of all studies meeting the inclusion criteria were reviewed to identify other potentially eligible studies not indexed in the databases.

### Search methods for identification of studies

The search terms included ‘Anterior Cruciate Ligament’, ‘four strands’, ‘five strands’, ‘six strands’ and ‘hamstrings’. The full PubMed search strategy is presented in Supporting Information S1: File 1. Two independent reviewers screened the titles and abstracts of all articles retrieved from the search, based on the eligibility criteria. Any disagreements between the reviewers regarding study inclusion were resolved through discussion with a third reviewer until a consensus was reached.

### Data extraction and data items

Data were extracted independently by two reviewers, and discrepancies were resolved by consensus with a third reviewer. The following characteristics were extracted from the included studies: study, country of origin, study period, duration of follow‐up, study design, number of patients enroled, mean patient age and proportion of females. Additional information extracted when available included sources of funding or conflicts of interest. Comparable outcomes extracted included graft characteristics, such as anterior laxity measured by KT‐1000/2000 arthrometers and graft diameter. Patient‐reported outcomes compiled included the Lysholm score, Tegner activity score, International Knee Documentation Committee (IKDC) subjective knee form score, Knee Injury and Osteoarthritis Outcome Score (KOOS) subscales, and physical and mental component summaries of the Short Form 36 health survey. The clinical examination findings captured were Lachman and pivot‐shift tests graded from 0 to 3, and hop test distance (participants performed three consecutive hops in a straight line for maximal distance). Graft failure incidence was also extracted, as previously reported.

### Assessment of risk of bias in included studies

The risk of bias for the included randomized controlled trials was assessed independently by two reviewers using the Cochrane Collaboration tool. It evaluates six domains: randomization process, deviations from intended interventions, missing outcome data, measurement of outcome, selection of reported results, and other biases. Each domain was assigned a low‐, high‐ or unclear risk rating. These evaluations are presented in Supporting Information S1: File 2.

For nonrandomized studies, the Methodological Index for Non‐Randomized Studies (MINORS) was used to critically appraise quality based on 12 predefined items [16]. For noncomparative studies, total scores ranging from 0 to 4 were considered very low quality, to 5–7 as low quality, to 8–12 as fair quality, and 13 or higher as high quality. Comparative studies were evaluated on a more stringent scale, with scores of 0–6 deemed very low quality, 7–10 as low quality, 11–15 as fair quality, and 16 or above representing high quality [17].

### Assessment of results

RevMan 5.4 statistical software was used for data synthesis and analysis. Dichotomous outcomes were expressed as odds ratios (ORs) with 95% confidence intervals (CIs). Continuous outcomes were measured using either the mean difference (MDs) or standardized mean difference (SMDs) when different scales were used across studies to measure the same outcome. Associated 95% CIs were calculated as appropriate. Heterogeneity between studies was evaluated using the chi‐square test and *I* ^2^ statistics, with *I* ^2^ values of 25%, 50% and 75% representing low, moderate, and high heterogeneity, respectively. A fixed‐effects model was used if heterogeneity was negligible (*I* ^2^ < 75%); otherwise, a random‐effects model was applied. Missing or unclear data were handled according to the guidelines outlined in the Cochrane Handbook for Systematic Reviews of Interventions [5].

### Risk of bias across the studies

Publication bias was assessed using funnel plots, where the standard error of the effect estimate for each study was plotted against its effect size. Funnel plots were generated using Review Manager 5.4 software, with effect estimates plotted on the *x*‐axis and their corresponding standard errors on the *y*‐axis. Visual inspection of funnel plot asymmetry was used to evaluate possible publication bias in a way that asymmetry may suggest a lack of published or located studies. A symmetrical inverted funnel shape indicates an absence of bias, whereas asymmetry can arise if smaller studies showing no effect or even an increased risk with the intervention are less likely to be published than larger studies showing beneficial or significant effects.

### Additional analyses

Prespecified subgroup analyses were conducted based on the type of graft strand used (e.g., 4‐strand vs. other strand configurations).

Sensitivity analyses were performed to assess the robustness of the primary findings. This involved re‐analyzing the data by sequentially removing the highest‐weighted study from the meta‐analysis and observing the influence on the overall effect estimates.

The quality of evidence for each outcome was evaluated using the GRADE approach, which considers the risk of bias, consistency, directness, precision of estimates and potential reporting biases. These principles rate the quality of evidence from randomized trials as high, moderate, low or very low to guide clinical recommendations [6].

## RESULTS

### Study selection

**Figure 1:**
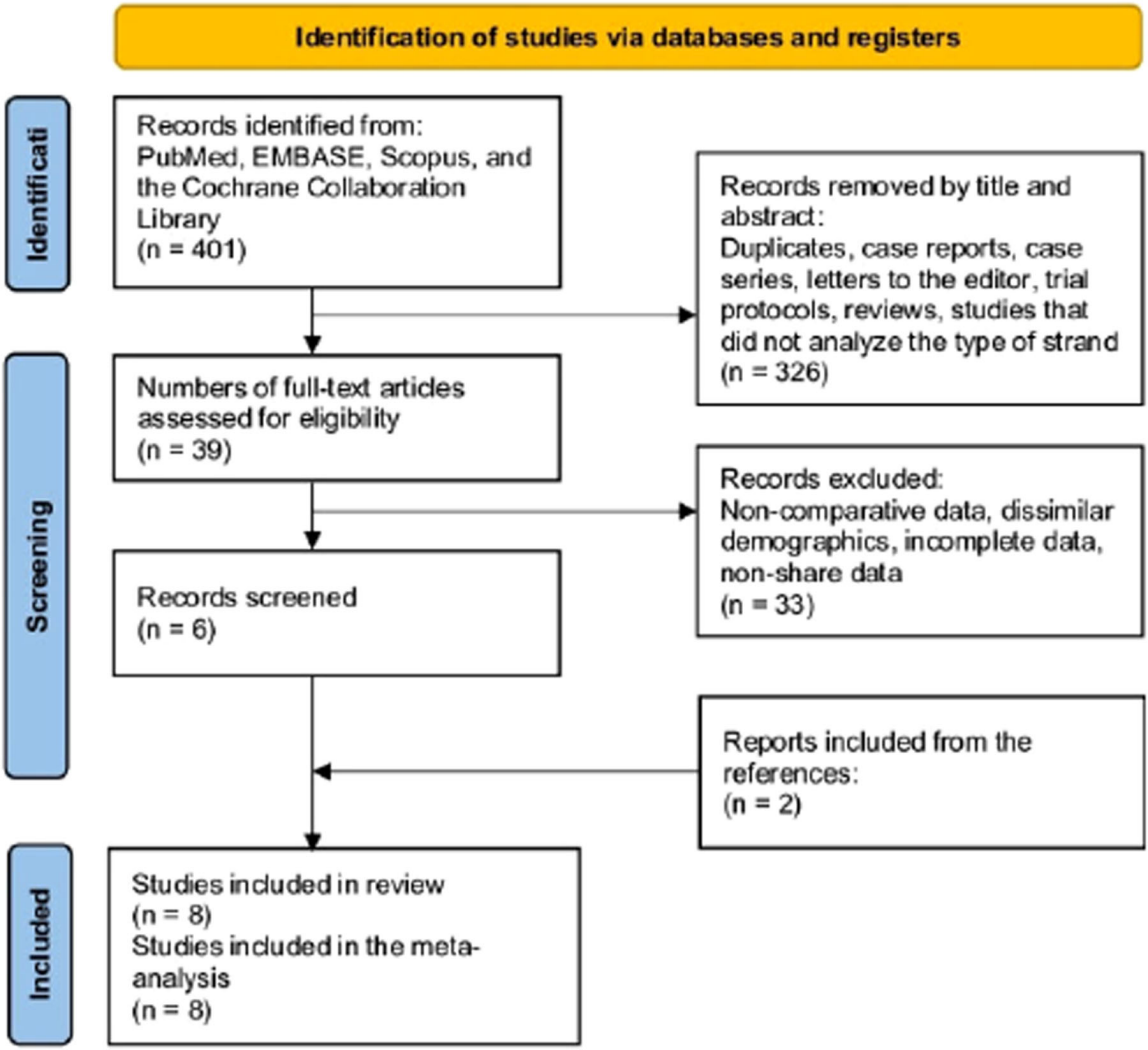
PRISMA guidelines for this research.

The systematic literature search yielded 401 records that were screened independently by two reviewers according to the predefined eligibility criteria (JMF and ME). Titles and abstracts were first assessed, and 326 records were excluded for duplicates, case reports/series, letters, protocols, reviews or noncomparative studies that did not analyze specific graft strands. The full texts of the remaining 39 articles were retrieved and evaluated in detail, leading to the exclusion of a further 33 studies for reasons such as noncomparative data collection, dissimilar patient characteristics between groups or incomplete/unavailable outcome reporting. This process identified six studies that initially met all inclusion criteria for qualitative synthesis. Two additional relevant studies were identified by screening their reference lists of included studies. Eight comparative studies were ultimately eligible and included in the quantitative meta‐analysis [3, 7, 9, 10, 11, 15] (Figure 1).

### Risk of bias

**Figure 2:**
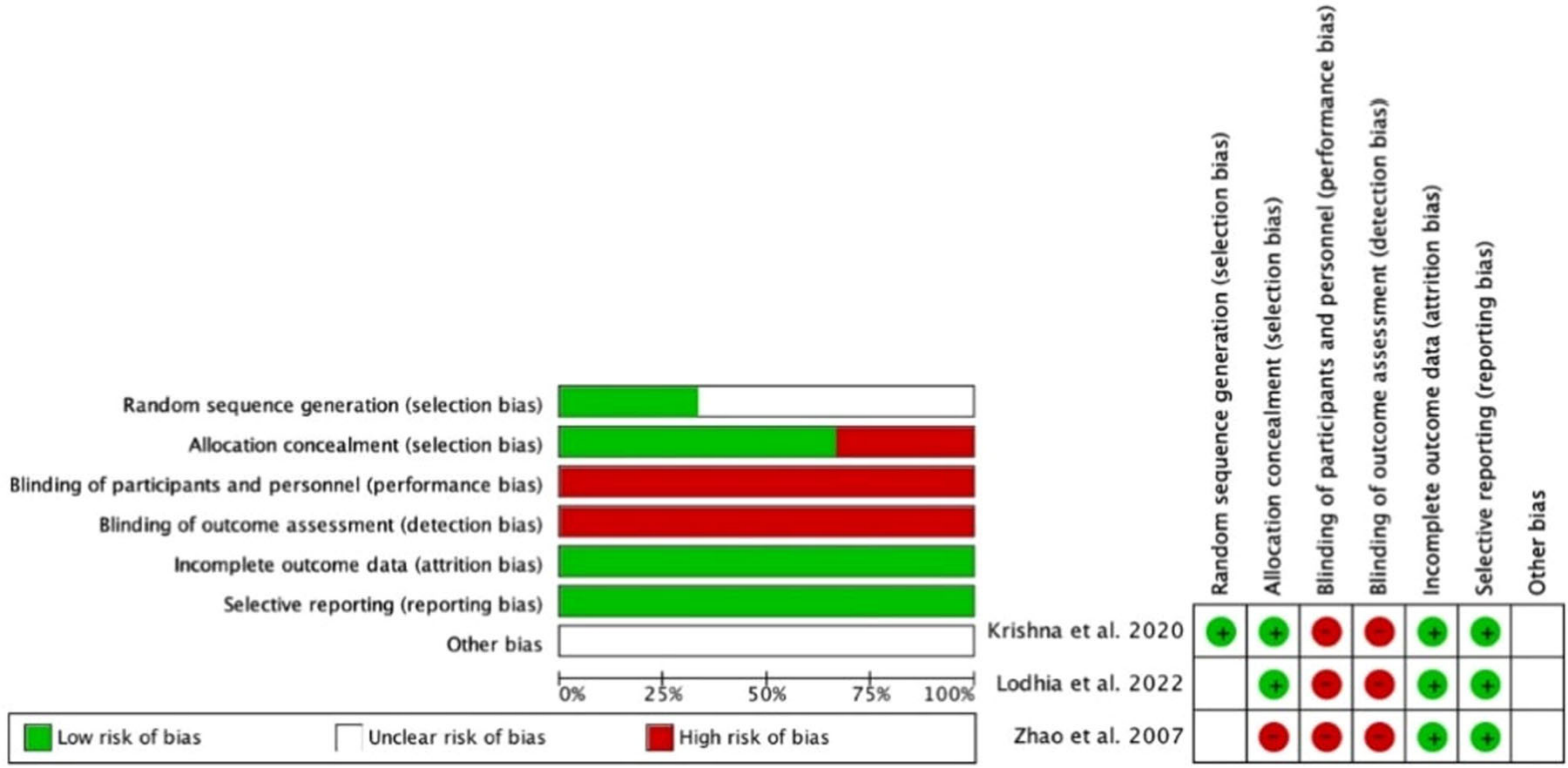
Assessment of risk of bias in included studies with six different domains.

The risk of bias for the included randomized controlled trials was moderate (Figure 2). Blinding of participants, personnel and outcome assessment was not possible because of the surgical nature of the interventions, potentially introducing performance and detection bias. However, the studies appeared to have adequately reported all prespecified outcomes and provided reasons for any participant loss or withdrawal during follow‐up.

For the nonrandomized comparative studies, the methodological quality was high in five of the six studies and fair quality in one of the six studies included according to the MINORS criteria (Table 1). These studies scored well across items related to a clearly stated aim, inclusion of consecutive patients, endpoints appropriate to the aim of the study, unbiased assessment of the study endpoint, follow‐up period appropriate to the aim of the study, and prospective calculation of the study size. The remaining comparative studies were deemed to have acceptable methodological quality based on clear reporting of the included populations, interventions, and study endpoints. The main limitation of the nonrandomized evidence was that not all studies adequately reported losses to follow‐up or reasons for missing outcome data.

**Table 1:** Assessment of the quality of studies through Methodological Index for Non‐Randomized Studies (MINORS).

Study	Clearly stated aim	Consecutive patients	Prospective collection data	Endpoints	Assessment endpoint	Follow‐up period	Loss less than 5%	Study size	Adequate control group	Contemporary group	Baseline control	Statistical analyses	MINORS
**Attia et al.** [1]	2	2	2	2	2	2	0	2	2	2	2	2	22
**Calvo et al. 2016**	2	2	0	1	2	2	0	2	2	2	2	2	19
**Ignozzi et al.** [7]	2	2	0	2	2	2	0	2	2	2	2	2	20
**Krishna et al.** [10]	2	2	0	2	2	2	0	2	2	2	2	2	20
**Sideris et al. 2017**	2	2	2	2	2	1	0	1	1	2	1	2	18

### Study characteristics

Table 2 presents the baseline characteristics of the included studies. Eight studies with a total of 1161 participants were included. The 4‐strand group consisted of 412 participants, whereas the control group (different from 4‐strand) consisted of 749 participants. Five of the studies were cohort studies (four retrospective and one prospective), whereas three were randomized controlled trials. The follow‐up period varied between 12.0 weeks and 44.3 months. The mean age in the 4‐strand group ranged from 19.0 to 32.1 years, while in the control group it ranged from 18.7 to 32.4 years. The numbers of female participants, conflicts of interest, and funding sources are listed in Table 2.

**Table 2:** Baseline characteristics of the eight included studies.

Study	Region	Period	Follow‐up (months)	Study design	n 4‐Strand	n Control	Age 4‐Strand	Age Control	Female 4‐Strand	Female control	Conflict of interest (Yes/No)	Funding (Yes/No)
**Attia et al.** [1]	Qatar	2011–2017	44.3	Retrospective cohort	33	5‐Strand: 224; 6‐Strand: 156	32.1	5‐Strand: 30.9; 6‐Strand: 32.4	2	5‐Strand: 4; 6‐Strand: 4	No	NR
**Calvo et al. 2016**	Chile	2012–2013	31.3	Retrospective cohort	33	5‐Strand: 37	29.7	5‐Strand: 30.2	NR	NR	Yes	NR
**Ignozzi et al.** [7]	USA	2013–2018	37.8	Retrospective cohort	51	5‐Strand: 23	31.8	5‐Strand: 23.6	23	5‐Strand:11	Yes	No
**Krishna et al.** [10]	Singapore	2014–2016	17.3	Retrospective cohort	20	5‐Strand: 25	29.2	5‐Strand: 25.6	1	5‐Strand: 6	No	NR
**Krishna et al. 2020**	Singapore	2015–2017	24.0	RCT	28	5‐Strand: 28	27.6	5‐Strand: 26.3	8	5‐Strand: 5	NR	NR
**Lodhia et al.** [11]	Canada	NR	24.0	RCT	191	5‐Strand: 208	19.0	5‐Strand: 18.7	63	5‐Strand: 108	Yes	No
**Sideris et al. 2017**	Australia	2014	12.0^a^	Prospective cohort	18	5‐Strand: 10	29.3	5‐Strand: 26.7	10	5‐Strand: 1	NR	NR
**Zhao et al.** [19]	China	2001–2002	35.0	RCT	38	8‐Strand:38	25.0	8‐Strand: 24.0	17	8‐Strand: 15	No	NR

Abbreviations: N/A, not applicable; NR, not reported; RCT, randomized clinical trial.

a Weeks.

### Graft characteristics

**Figure 3:**
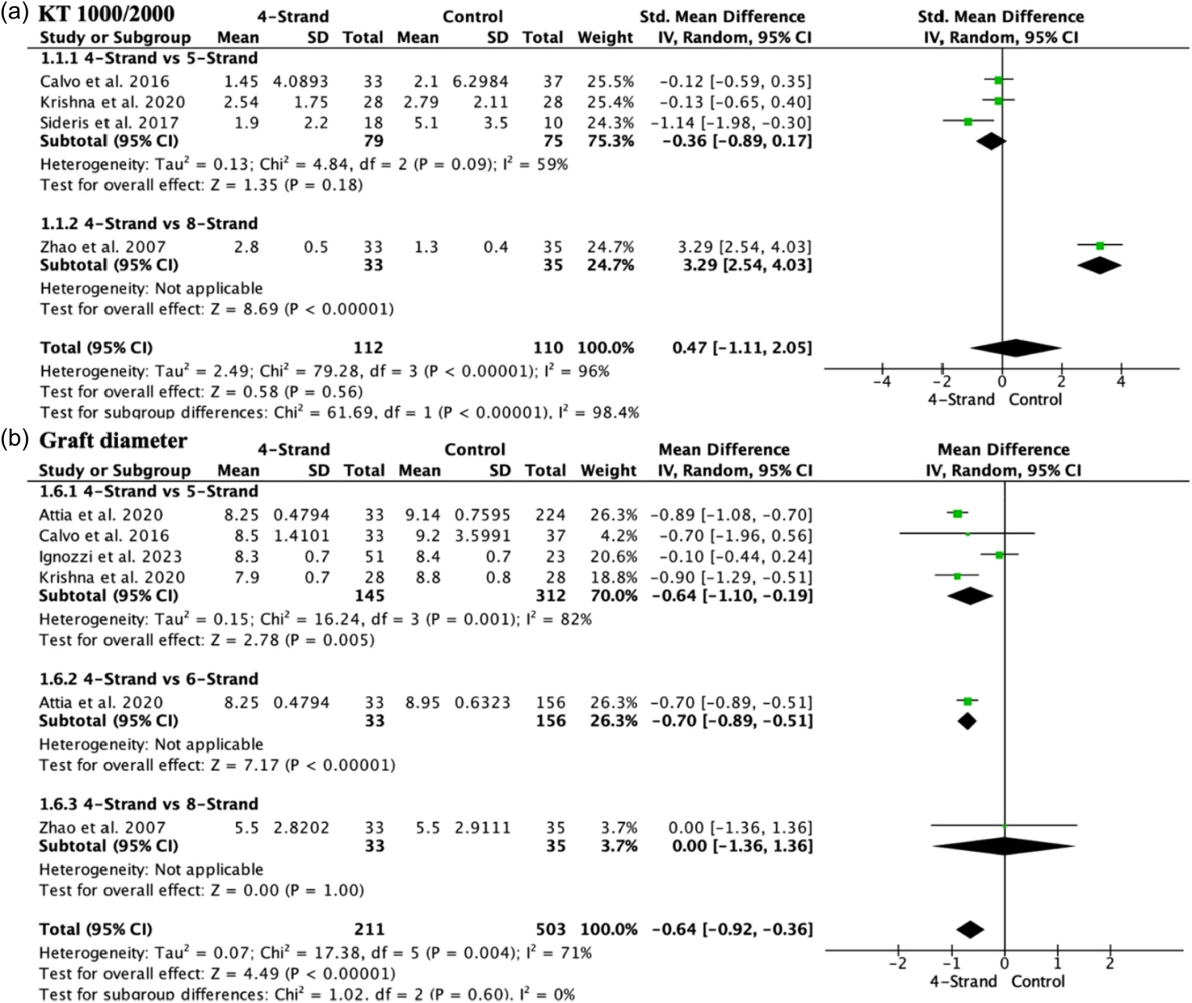
Forest plot of graft characteristics. (a) laxity measured by KT1000 and KT2000 (b) graft diameter.

Regarding the average laxity measured by KT1000 and KT2000, no significant differences were found (SMD 0.47, 95% CI −1.11 to 2.05; participants = 222; studies = 4; *I* ^2^ = 96%) (Figure 3a). When subgroup analyses were conducted, no significant differences were found when comparing four versus five strands (SMD: −0.36, 95% CI −0.89, 0.17; participants = 154; studies = 3; *I* ^2^ = 59%). When comparing 4 versus 8 strands, the 8‐strand group showed significantly lower scores on the KT1000 (SMD 3.29, 95% CI 2.54–4.03; participants = 68; studies = 1; *I* ^2^ = 0%), although only one study evaluated this subgroup. Graft diameter was significantly smaller in the 4‐strand group (MD −0.64, 95% CI −0.92 to −0.36; participants = 714; studies = 6; *I* ^2 ^= 71%) (Figure 3b). When subgroup analyses were performed, the 4‐strand group had a significantly smaller graft diameter than the 5‐strand (MD −0.64, 95% CI −1.10 to −0.19; participants = 457; studies = 4; *I* ^2^ = 82%) and 6‐strand (MD −0.70, 95% CI −0.89 to −0.51; participants = 189; studies = 1; *I* ^2^ = 0%) groups. No differences were found between the 4‐strand and 8‐strand groups (MD 0.00, 95% CI −1.36 to 1.36; participants = 68; studies = 1; *I* ^2^ = 0%).

### PROMs (Patient‐Reported Outcome Measures)

**Figure 4:**
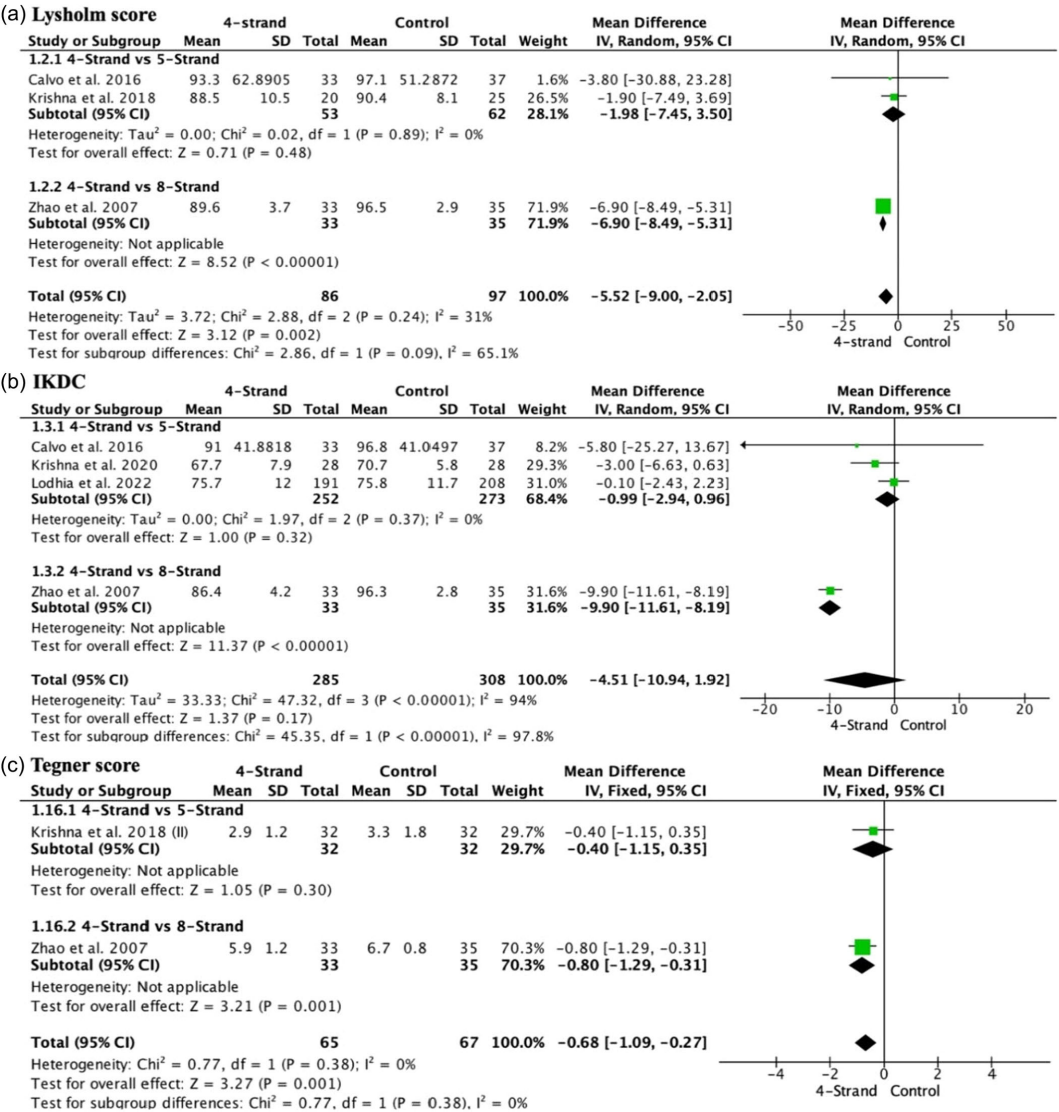
Forest plot of patient‐reported outcome measures (PROMs). (a) Lysholm score. (b) (International Knee Documentation Committee (IKDC). (c) Tegner score.

The Lysholm score was lower in the 4‐strand group (MD −6.52, 95% CI −8.04 to −5.00; participants = 183; studies = 3; *I* ^2^ = 31%) (Figure 4a). No significant differences were found when compared to 5‐strand (MD −1.98, 95% CI −7.45 to 3.50; participants = 115; studies = 2; *I* ^2^ = 0%), but significantly lower values were observed compared to 8‐strand (MD −6.90, 95% CI −8.49 to −5.31; participants = 68; studies = 1; *I* ^2^ = 100%). IKDC did not show significant differences among the groups overall (MD −4.51, 95% CI −10.94 to 1.92; participants = 593; studies = 4; *I* ^2^ = 94%) (Figure 4b). When subgroup analyses were conducted, no significant differences were found when comparing 4 versus 5‐strand (MD −0.99, 95% CI −2.94 to 0.96; participants = 525; studies = 3; *I* ^2^ = 0%), but there were differences observed with lower values in the 4‐strand group (MD −9.90, 95% CI −11.61 to −8.19; participants = 68; studies = 1; *I* ^2^ = 0%). The Tegner score showed significantly lower values in the 4‐strand group overall (MD −0.68, 95% CI −1.09 to −0.27; participants = 132; studies = 2; *I* ^2^ = 0%) (Figure 4c). No significant differences were found when comparing 4‐ versus 5‐strand (MD −0.40, 95% CI −1.15 to 0.35; participants = 64; studies = 1; *I* ^2^ = 0%), but significant differences were observed with lower values in the 4‐strand group compared to the 8‐strand (MD −0.80, 95% CI −1.29 to −0.31; participants = 68; studies = 1; *I* ^2^ = 0%). Table 3 presents the results of the KOOS and SF‐36 scales. No significant differences were found between the 4‐strand and 5‐strand.

**Table 3:** KOOS and SF‐36 assessment at final follow‐up.

Effect size	*n* studies	*n* participants	Fixed effect model (MD 95% CI)	*I* ^2^ (%)	*p* value
KOOS Symptoms	2	101	MD −3.17, 95% CI −7.78 to 1.44	66%	*p* = *0.18*
KOOS Pain	2	101	MD −1.12, 95% CI −3.44 to 1.20	0%	*p* = 0.58
KOOS Sports	2	101	MD −2.98, 95% CI −9.33 to 3.36	0%	*p* = 0.36
SF‐36 PCS	2	101	MD 0.67, 95% CI −1.23 to 2.56	0%	*p* = 0.49
SF‐36 MCS	2	101	MD 1.18, 95% CI −1.41 to 3.78	51%*	*p* = 0.37

Abbreviations: CI, confidence interval; KOOS, Knee Injury and Osteoarthritis Outcome; MD, mean difference; SF‐36, Short Form 36.

*p < 0.05.

### Failure

**Figure 5:**
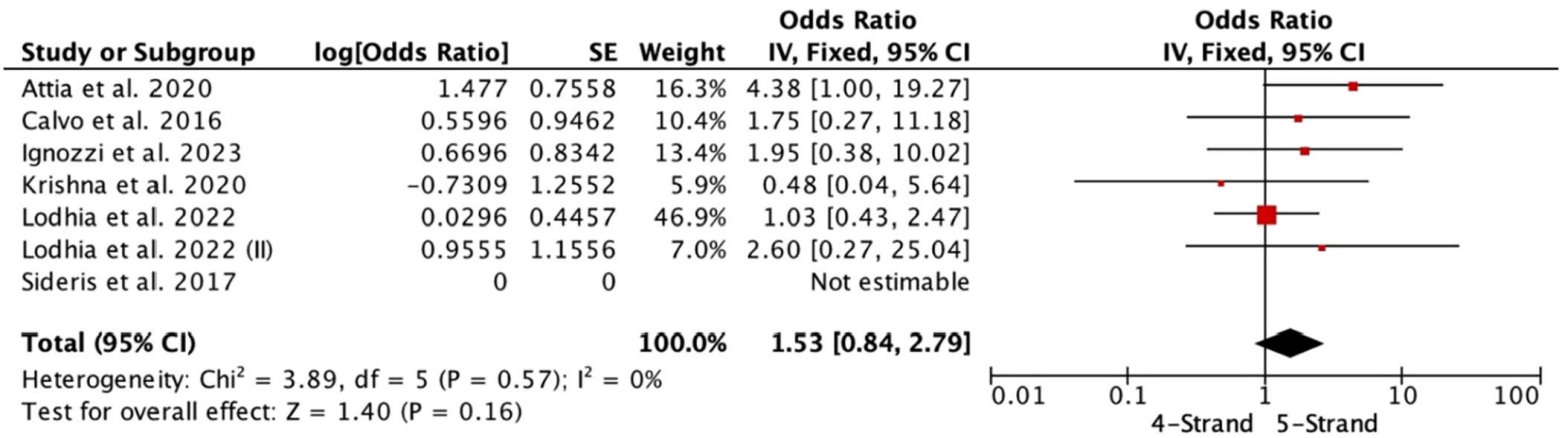
Forest plot about graft failure.

Graft failure did not show significant differences when comparing 4‐strand versus 5‐strand (OR 1.53, 95% CI 0.84–2.79; studies = 7) (Figure 5).

### Clinical tests

There were no significant differences in the clinical tests assessed by the Lachman test, pivot‐shift test and hop test (Table 4).

**Table 4:** Clinical test at final follow‐up.

Effect size	*n* studies	*n* participants	Fixed effect model (OR/MD 95% CI)	*I* ^2^ (%)	*p* value
Lachmann 0	2	455	OR 1.07, 95% CI 0.74–1.55	0%	*p* = 0.71
Lachmann 1	2	455	OR 1.03, 95% CI 0.68–1.56	0%	*p* = 0.89
Lachmann 2	2	455	OR 0.35, 95% CI 0.11–1.09	0%	*p* = 0.07
Lachmann 3	2	455	OR 0.36, 95% CI 0.01–8.92	0%	*p* = 0.53
Pivot‐shift 0	2	455	OR 0.99, 95% CI 0.69–1.44	0%	*p* = 0.97
Pivot‐shift 1	2	455	OR 0.90, 95% CI 0.58–1.39	17%	*p* = 0.64
Pivot‐shift 2	2	455	OR 1.25, 95% CI 0.43–3.64	0%	*p* = 0.68
Pivot‐shift 3	2	455	N/E	N/E	N/E
Hop test	2	130	MD −0.25, 95% CI −3.79–3.28	0%	*p* = 0.89

Abbreviations: CI, confidence interval; MD, mean difference; N/E, not estimable; OR, odds ratio.

### Publication bias

**Figure 6:**
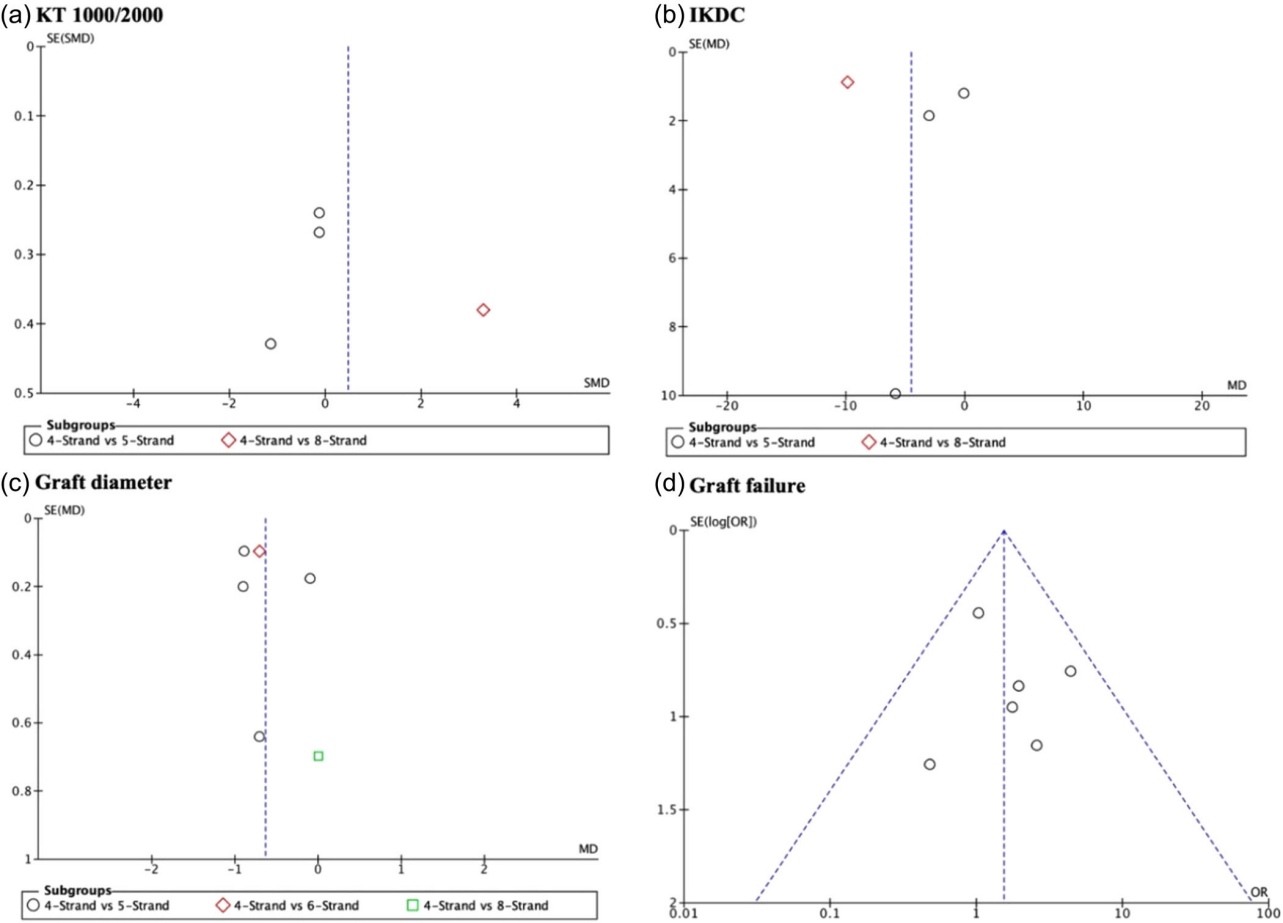
Funnel plot of the study. (a) laxity by KT1000 and KT2000. (b) IKDC (c) Graft diameter (d) Graft failure.

Publication bias (Figure 6) was present for the variables KT 1000/2000, IKDC, and graft diameter, indicating a potential bias towards publishing studies with significant results. No publication bias was observed for graft failure. Owing to the limited number of studies available for the remaining variables, it was not possible to assess publication bias more consistently.

### Additional analyses

Sensitivity analysis, which involved removing the study with the highest weight, did not change the direction of any of the results.

### GRADE

In relation to the most relevant outcomes, graft failure showed moderate certainty, whereas IKDC demonstrated low certainty. Measurement of graft characteristics, such as graft diameter and KT 1000/2000, exhibited low and very low certainty, respectively (Table 5). The studies primarily lacked nonrandomized designs and there was a high risk of publication bias. Additionally, clinical differences, including variations in study types and number of strands used, contributed to the lack of consistency in the results.

**Table 5:** GRADE assessment of the quality of the evidence and the strength of the recommendations.

Certainty assessment							№ of patients		Effect		Certainty	Importance
№ of studies	Study design	Risk of bias	Inconsistency	Indirectness	Imprecision	Other considerations	Outcomes	placebo	Relative (95% CI)	Absolute (95% CI)		
**KT 1000/2000**												
4	Nonrandomised studies	Not serious	Serious^a^	Serious^b^	Not serious	Publication bias strongly suspected	112	110	‐	SMD 0.47 higher	⊕○○○	CRITICAL
										(1.11 lower to 2.05 higher)	Very low	
**IKDC**												
4	Randomised trials	Serious	Not serious	Serious^b^	Serious^c^	Publication bias strongly suspected	285	308	‐	MD 4.51	⊕⊕○○	CRITICAL
										lower (10.94 lower to 1.92 higher)	Low	
**Graft failure**												
7	Nonrandomised studies	Not serious	Not serious	Serious^b^	Not serious	Publication bias not suspected	‐/0	‐/0	OR 1.53	2 fewer per 1000	⊕⊕⊕○	CRITICAL
									(0.84–2.79)	(from 3 fewer to 1 fewer)	Moderate	
**Graft diameter**												
5	Nonrandomised studies	Not serious	Not serious	Serious^b^	Not serious	Publication bias strongly suspected	211	503	‐	MD 0.64 lower	⊕⊕○○	IMPORTANT
										(0.92 lower to 0.36 lower)	Low	

Abbreviations: CI, confidence interval; MD, mean difference; OR, odds ratio; SMD, standardised mean difference.

c. Publication bias assessed by funnel plot.

^a^ Results showed wide variability or unexplained heterogeneity.

^b^ Clinical differences such as the type of study and the strands used.

^c^ Wide confidence intervals.

## DISCUSSION

The purpose of this meta‐analysis was to evaluate how the number of strands in graft configuration affects the clinical outcomes of ACL reconstruction. The primary hypothesis of this study was that, compared to the commonly used four‐strand grafts, those composed of five or more strands would provide greater strength and improved clinical outcomes. Although our findings offer some support for this hypothesis, the relationship between graft configuration and long‐term functional recovery proved to be more complex than initially expected.

The number of strands in graft design directly impacts graft diameter, stiffness, and resistance to elongation under load. Theoretically, grafts with larger diameters, higher tensile strength, and lower laxity should lead to greater joint stability and a reduced failure rate. Studies have shown that hamstring autografts with diameters smaller than 8 mm are significantly associated with an increased risk of failure, particularly in young, active populations [13]. In our research, grafts with five‐ or eight‐strand configurations tended to achieve larger diameters, frequently exceeding the critical threshold of 8 mm. These findings support the hypothesis that a higher number of strands can improve the mechanical robustness of the graft.

In this context, the concept ‘superior biomechanical properties’ include higher maximum load to failure, improved stiffness, reduced anterior tibial translation (measured with instrumented devices), and a larger cross‐sectional area. These mechanical benefits are particularly important during the early stages of graft incorporation, when elongation and micromotion may compromise healing. According to the results of the included studies, multistrand configurations consistently outperformed four‐strand grafts in these mechanical metrics, likely contributing to greater graft longevity.

However, while grafts with more strands demonstrated superior mechanical stability, this did not always translate into significantly better clinical outcomes when assessed using standardized tools such as the Lysholm Knee Score or the subjective International Knee Documentation Committee (IKDC) score. These results raise questions about the clinical applicability of biomechanical superiority, especially considering that patient‐centred outcomes are the gold standard for surgical success.

This discrepancy is not new. Although graft mechanics are crucial, the overall effectiveness of ACL reconstruction is influenced by a complex interplay of biological, behavioural, and technical factors, as shown in previous studies by Noyes and Barber‐Westin [2]. Subjective and objective outcomes are heavily affected by age, sex, preinjury activity level, graft healing, neuromuscular recovery and adherence to postoperative rehabilitation programs. Therefore, even in cases where a graft has superior mechanical properties, patient‐specific factors may overshadow any potential advantages conferred by the number of strands.

Interestingly, our meta‐analysis also found that revision rates between four‐ and five‐strand grafts did not differ significantly across several studies. This observation suggests that other factors—such as surgical technique, tunnel positioning and fixation method—may play a more decisive role in preventing graft failure than graft size alone [4]. For instance, improper tunnel placement has been cited as a major cause of ACL reconstruction failure, regardless of graft type or size [8]. Additionally, the fixation method used (e.g., suspensory fixation vs. interference screw) can alter initial graft tension and affect its early mechanical behaviour.

Another important factor to consider is the increasing use of allografts in ACL repair. Allografts offer practical benefits, such as shorter surgical times and the elimination of donor site morbidity. However, several studies have shown that allografts are associated with higher failure rates and delayed biological incorporation, particularly in young, active patients [19]. For this reason, our meta‐analysis primarily focused on autografts to ensure reliability and comparability of results. Nonetheless, including allografts in future research could provide a broader understanding of how strand configuration affects different graft types [12, 18].

High‐quality, multicenter randomized controlled trials that directly compare various hamstring graft topologies using standardized protocols for fixation, rehabilitation, and surgical technique are obviously needed. In order to assess graft longevity and the onset of osteoarthritis, a recognized consequence after ACL restoration, such studies ought to include longer‐term follow‐up.

Future studies should also examine the return‐to‐sport schedules, patient satisfaction, and cost‐effectiveness of various graft architectures. Comparative efficacy study should broaden to incorporate synthetic grafts and quadriceps tendon autografts, as the options for ACL grafts become more varied, in relation to hamstring strand configurations.

Our findings suggest that, from a surgical planning perspective, augmentation techniques should be considered when a four‐strand graft results in a diameter smaller than 8 mm. This includes using different graft sources, combining autograft and allograft tissue, or increasing the number of strands using additional autologous tissue. In such cases, five‐ or six‐strand configurations may offer a viable and biomechanically favourable solution, particularly in smaller patients or when graft availability is limited.

## CONCLUSION

This meta‐analysis suggests that higher strand numbers in hamstring autografts for ACL reconstruction—particularly 5‐ and 8‐strand configurations—may offer biomechanical and functional advantages over the traditional 4‐strand approach, particularly in graft diameter and selected patient‐reported outcomes. However, due to heterogeneity and limited data, especially for 6‐ and 8‐strand grafts, long‐term data would be necessary to fully understand the impact of graft configuration on joint health, function, and osteoarthritis development.

## LIMITATIONS

This meta‐analysis is subject to several limitations. The significant difference in surgical technique, graft source, fixation methods, and rehabilitation strategies among the included studies may have introduced confounding factors. Additionally, many trials only reported short‐ to mid‐term findings, and follow‐up periods varied, which reduced the ability to draw conclusions on long‐term effects like the beginning of osteoarthritis. The lack of individual patient data prevented more comprehensive analysis by age, sex, BMI or level of exercise before to injury. Furthermore, crucial surgical factors including the kind of femoral fixation and the tunnel's length were unknown.

Another methodological issue was the variability of outcome measures; not all studies consistently used standardized scales like IKDC or Lysholm or offered objective laxity assessments, making it difficult to draw trustworthy comparisons. Lastly, the statistical power to identify clinically meaningful changes may have been hampered by the small sample sizes in several investigations.

Publication bias is a potential limitation of this review, as studies with positive or significant results are more likely to be published than those with negative or inconclusive findings. This can lead to an overestimation of the true effect size. Although we attempted to minimize this risk by conducting a comprehensive literature search across multiple databases and by screening reference lists, the possibility of publication bias cannot be entirely excluded. Therefore, the results should be interpreted with caution, considering this potential source of bias.

## AUTHOR CONTRIBUTIONS

All authors contributed to the study conception and design. Material preparation, data collection and analysis were performed by Juan Manuel Fernández Domínguez, Jose Luis Martín Alguacil, Marta Esteban Blanco, Manuel Vides Fernández, Joan Carles Monllau.

## CONFLICT OF INTEREST STATEMENT

The authors declare no conflicts of interest.

## ETHICS STATEMENT

The authors have nothing to report.

## Supporting information


**Supplementary File 1.** PubMed search strategy.

**Supplementary file 2.** Risk of bias judgement.

## Data Availability

The data that support the findings of this study are available from the corresponding author upon reasonable request.
