# Supplementary material for: A meta‐analysis of the hamstring tendon strands reconstruction in ACL: Functional outcomes based on strands number
Source: J Exp Orthop. 2025 Nov 3;12(4):e70508. doi: 10.1002/jeo2.70508 (PMC12581842; doi:10.1002/jeo2.70508)
Supplement: Supplementary file 1 — PubMed search strategy. [file JEO2-12-e70508-s001.docx]

**Supplementary File 1.** PubMed search strategy.

Search: **("Anterior Cruciate Ligament" OR ACL) AND ( "four strand" OR quadruple OR 4-strand OR "five strand" OR 5-strand OR "six strand" OR 6-strand) AND hamstring**

("Anterior Cruciate Ligament"[All Fields] OR ("Anterior Cruciate Ligament"[MeSH Terms] OR ("anterior"[All Fields] AND "cruciate"[All Fields] AND "ligament"[All Fields]) OR "Anterior Cruciate Ligament"[All Fields] OR "acl"[All Fields])) AND ("four strand"[All Fields] OR ("quadruple"[All Fields] OR "quadruples"[All Fields]) OR "4-strand"[All Fields] OR "five strand"[All Fields] OR "5-strand"[All Fields] OR "six strand"[All Fields] OR "6-strand"[All Fields]) AND ("hamstring muscles"[MeSH Terms] OR ("hamstring"[All Fields] AND "muscles"[All Fields]) OR "hamstring muscles"[All Fields] OR "hamstring"[All Fields] OR "hamstrings"[All Fields])

**Translations**

**ACL:** "anterior cruciate ligament"[MeSH Terms] OR ("anterior"[All Fields] AND "cruciate"[All Fields] AND "ligament"[All Fields]) OR "anterior cruciate ligament"[All Fields] OR "acl"[All Fields]

**quadruple:** "quadruple"[All Fields] OR "quadruples"[All Fields]

**hamstring:** "hamstring muscles"[MeSH Terms] OR ("hamstring"[All Fields] AND "muscles"[All Fields]) OR "hamstring muscles"[All Fields] OR "hamstring"[All Fields] OR "hamstrings"[All Fields]
