# Supplementary material for: A meta‐analysis of the hamstring tendon strands reconstruction in ACL: Functional outcomes based on strands number
Source: J Exp Orthop. 2025 Nov 3;12(4):e70508. doi: 10.1002/jeo2.70508 (PMC12581842; doi:10.1002/jeo2.70508)
Supplement: Supplementary file 2 — Risk of bias judgement. [file JEO2-12-e70508-s002.docx]

**Supplementary file 2.** Risk of bias judgement.

**Krishna et al. 2020**

A) Block randomization was performed using an online randomization generator 144 (http://www.graphpad.com/quickcalcs). The patients were randomized into 2 groups – ACL 145 reconstruction with 5-strand (n=32, study group) or 4-strand (n=32, control group) hamstring 146 autograft (Fig 1). We utilized an intention-to-treat approach in our prospective study.

B) Figure 1.

C) There was no blinding

D) There was no blinding.

E) Figure 1.

F) All kind of outcomes.

**Lodhia et al. 2022**

A) Not reported.

B) Figure 1.

C) Not reported.

D) Not reported.

E) Figure 1.

F) All kind of outcomes.

**Zhao et al. 2007**

A) Not reported.

B) Not reported.

C) Not reported.

D) Not reported.

E) The reason for the lack of follow-up was a loss of contact in 3 patients and a communication problem in 5.

F) All kind of outcomes.
